# Bereavement, post-traumatic stress and post-traumatic growth: through the lenses of positive psychology

**DOI:** 10.1080/20008198.2017.1351220

**Published:** 2017-09-29

**Authors:** Olga O. Thomadaki

**Affiliations:** ^a^ Deree, The American College of Greece, Athens, Greece

**Keywords:** Perinatal bereavement, post-traumatic growth, interpretative phenomenological analysis, positive psychology

## Abstract

**Background**: During the past decades we have learned a lot about the psychopathology that can be triggered from adversity, but our knowledge of the qualities developed and processes experienced by people who remain healthy despite having gone through trauma has been limited. A proposed area within positive psychology is the psychology of loss. Psychology of loss emphasizes the study of healthy individuals, the resources they use for adapting to loss, and the ways in which they can transform their losses into personal growth and strength. Under the theoretical umbrella of positive psychology, the construct of post-traumatic growth has also received increased scholarly attention in recent years. Post-traumatic growth is a concept and a construct that reflects the ‘positive psychological change experienced as a result of the struggle with highly challenging life circumstances’ (Tedeschi & Calhoun, 2004). Numerous empirical studies have described the phenomenon after various traumas, including traumatic bereavement, even among children and adolescents (Kilmer et al., 2009).

**Objective**: The qualitative study presented was conducted in the UK, where eight women were interviewed after losing their first child perinatally. This type of bereavement constitutes a traumatic loss and although there is a plethora of research focusing on the resulting parental psychopathology, research on adaptive grief resolution and post-traumatic growth is scarce.

**Method**: The research question was ‘How mothers experience personal growth after a perinatal loss’ and the research methodology employed was interpretative phenomenological analysis (Smith, Flowers, & Larkin, 2009).

**Results**: The analysis revealed four superordinate themes. The first mainly presented the traumatic quality of this type of bereavement and included subthemes such as iatrogenic psychological trauma, while the second presented the multiple losses involved with emerging themes on the amputated object relationship, the socially unrecognized maternal identity and the threatened reproductive ability. The third superordinate theme presented all the coping mechanisms that were activated by participants to work through their loss; emerging themes were rumination and disillusionment, oscillating between action and avoidance, religious and spiritual coping, and the quality of social support as a catalyst. The fourth superordinate theme presented the positive changes that came as a consequence of the experience and their efforts to psychologically survive the loss. The subthemes that emerged in last superordinate theme were growth as an affirmation of the baby’s importance; growth as an outcome of the awareness of personal vulnerability; transformations in self-perception: self-worth and self-efficacy; transformations in perception and attitude: appreciation of life and changed priorities; and transformations in relationships: empathy and companionship.

**Conclusions**: The research findings suggest that following this traumatic loss, mothers, struggling with distress and anguish, can also experience positive transformations. The possible role of counselling psychologists and psychotherapists in this journey of personal positive transformation of bereaved individuals should be further explored and enhanced.
